# Transgenic overexpression of endogenous FLOWERING LOCUS T-like gene *MeFT1* produces early flowering in cassava

**DOI:** 10.1371/journal.pone.0227199

**Published:** 2020-01-28

**Authors:** John Odipio, Beyene Getu, R. D. Chauhan, Titus Alicai, Rebecca Bart, Dmitri A. Nusinow, Nigel J. Taylor

**Affiliations:** 1 Donald Danforth Plant Science Center, St. Louis, MO, United States of America; 2 National Crops Resources Research Institute, Kampala, Uganda; 3 Vlaams Instituut voor Biotechnologie, Department of Plant Biotechnology and Bioinformatics, Faculty of Sciences, Ghent University, Ghent, Belgium; National University of Kaohsiung, TAIWAN

## Abstract

Endogenous FLOWERING LOCUS T homolog *MeFT1* was transgenically overexpressed under control of a strong constitutive promoter in cassava cultivar 60444 to determine its role in regulation of flowering and as a potential tool to accelerate cassava breeding. Early profuse flowering was recorded *in-vitro* in all ten transgenic plant lines recovered, causing eight lines to die within 21 days of culture. The two surviving transgenic plant lines flowered early and profusely commencing as soon as 14 days after establishment in soil in the greenhouse. Both transgenic lines sustained early flowering across the vegetative propagation cycle, with first flowering recorded 30–50 days after planting stakes compared to 90 days for non-transgenic controls. Transgenic plant lines completed five flowering cycles within 200 days in the greenhouse as opposed to twice flowering event in the controls. Constitutive overexpression of *MeFT1* generated fully mature male and female flowers and produced a bushy phenotype due to significantly increased flowering-induced branching. Flower induction by *MeFT1* overexpression was not graft-transmissible and negatively affected storage root development. Accelerated flowering in transgenic plants was associated with significantly increased mRNA levels of *MeFT1* and the three floral meristem identity genes *MeAP1*, *MeLFY* and *MeSOC1* in shoot apical tissues. These findings imply that *MeFT1* encodes flower induction and triggers flowering by recruiting downstream floral meristem identity genes.

## Introduction

Millions of tropical households depend on cassava (*Manihot esculenta* Cranz) for food and economic security [1,2]. Farmers cultivate cassava for its starchy storage roots and utilize semi-woody stem cuttings as propagules to establish the next cropping cycle. As a result, selection for predictable and prolific flowering has not been prioritized during the crop’s domestication. Breeding programs dedicated to development of elite varieties to meet the needs of farmers and consumers require synchronized production of fertile flowers in desired parental lines. Such programs are, presently constrained by the genotype- and environment-dependent nature of flowering inherent to the crop [3,4]. Breeders address these challenges in several ways. Crossing blocks can be established in geographical regions that favour flowering, while varied planting times can help synchronize flowering of parental genotypes [5]. Foliar application of plant growth regulators [6] and grafting onto rootstocks obtained from high flowering varieties are also techniques employed to improve flowering and allow controlled breeding programs [4,7].

Temperature, day length (photoperiod), levels of sugar, nitrates and plant growth regulators have been identified as triggers for flowering in *Arabidopsis thaliana* and other plant species [8–10]. FLOWERING LOCUS T (FT) protein is synthesised in the leaf and is thought to be translocated via the phloem and through the graft unions to control flowering [11]. *FT* and other *FT*-like genes encoding phosphatidylethanolamine-binding proteins (PEBP) occur ubiquitously to regulate reproductive and physiological processes including storage organ development [12–14]. Upon delivery to the shoot apical meristematic (SAM) region, FT and the bZIP transcription factor FLOWERING LOCUS D (*FD*) form a protein complex which further interacts with SUPPRESSOR OF OVEREXPRESSION OF CONSTANS 1 (*SOC1*), APETALA 1 (*AP1*) and LEAFY (*LFY*) proteins to induce flowering [15–17]. Transition to reproductive development by *FT* upregulation have been associated with a measurable increase in mRNA levels of both *FT* and downstream floral meristem identity genes in the SAM [18–20].

Homologs of *FT* have been described outside Arabidopsis, including a recent report in cassava [21], with manipulation of *FT* gene expression resulting in precocious flowering [12,22,23]. For example, heterologous overexpression of Arabidopsis *FT* (*AtFT*) produced early, fertile flowers in citrus, poplar and cassava, overcoming juvenility, vernalization and photoperiod hurdles to flowering [22,24,25]. Transgenic potato and onion plants expressing *FT* also recorded improved flowering and storage organ development [14,18]. Functions of native *FT* genes in cassava remain largely unknown. Advances in genomics and biotechnology techniques such as transgenic and gene editing provide promise to address this challenge [7]. The objective of this study was to investigate the role of native *FT*-like gene *MeFT1* in regulation of cassava flowering and storage root development, thereby contributing towards an avenue for shortened breeding cycle and increased yield for this important staple crop.

## Materials and methods

### Selection of plant materials and target gene

The transformable variety 60444 [26] was selected for overexpression of *MeFT1*, and the lesser flowering cv. TME 204 was included for grafting investigations. A BLAST search of the cassava genome sequence in Phytozome version 4.1 (http://www.phytozome.net/) was conducted with Arabidopsis *FT* (AT1G65480.1) as a query sequence [21]. The first hit (Manes.12G001600.1) was named *MeFT1* after a BLAST query in NCBI confirmed it to be an *FT* gene. Amino acid sequences of both *MeFT1* and *AtFT* were subjected to pairwise alignment (https://www.ebi.ac.uk/Tools/psa/emboss_needle) to establish relationships between the two genes.

### Construction of *MeFT1* overexpression vector

**Fig 1:**
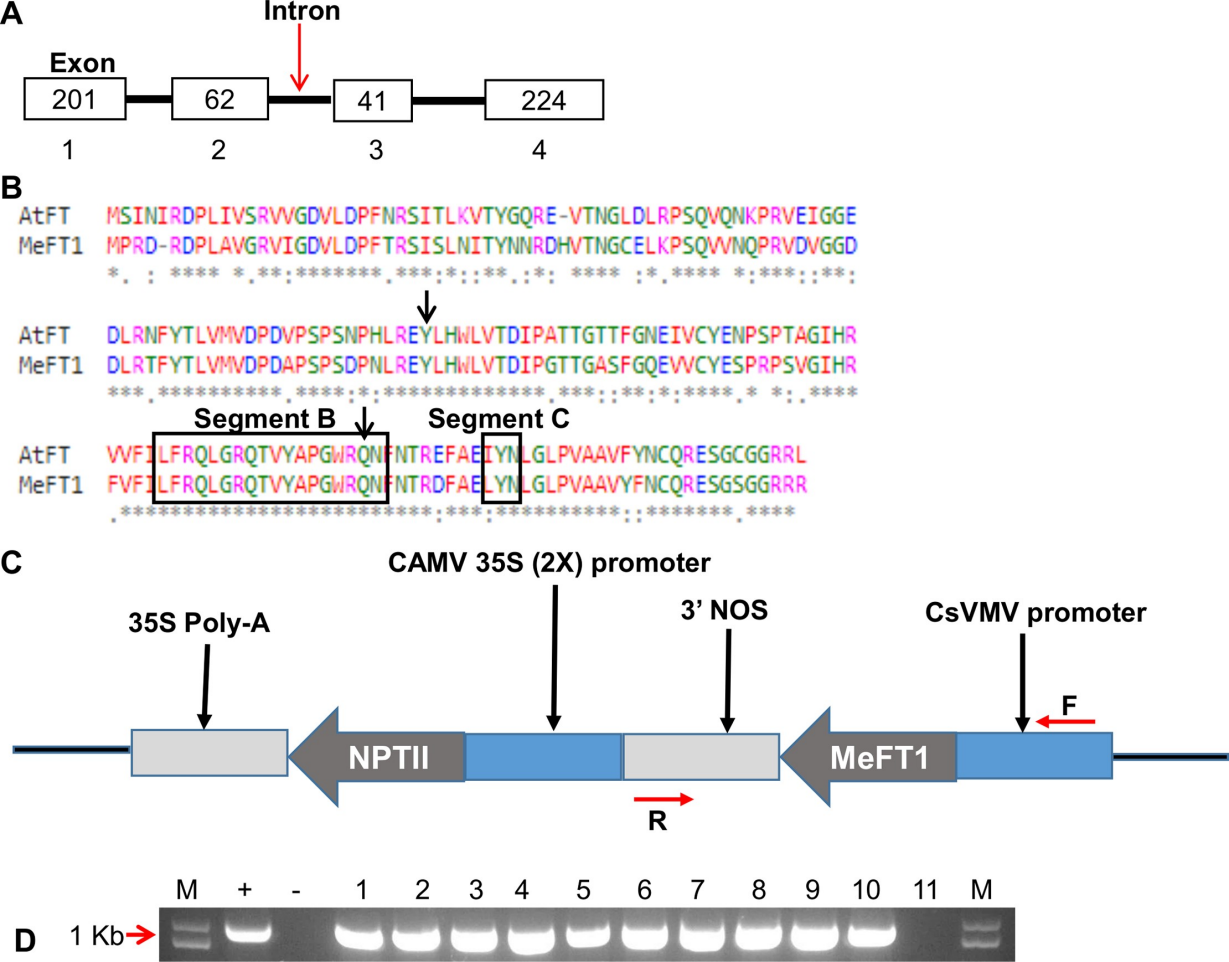
Characterization of *MeFT1* gene and detection of putative transgenic plant lines. (**A**) Genomic structure of *MeFT1* gene showing exons (box) and introns (line). Numbers in boxes represent size of exon (base pairs) and numbers below the box show exon number. (**B**) MUSCLE alignment of amino acid sequences from Arabidopsis *FT* and *MeFT1*. Boxes represent conserved sequences, black arrows represent critical amino acids that determine functionality of *FT* gene, Asterisk (*) indicates identical amino acids. (**C)** Linear vector map showing T-DNA used to overexpress *MeFT1* in transgenic cassava plants with *MeFT1* fused to the CsVMV promoter and NOS terminator. Neomycin phosphotransferase II (NPTII) confers paramomycin resistance for selection of transgenic cassava plants, **F and R** indicate forward and reverse primers used for PCR detection of the T-DNA cassette in transgenic plants). (**D**) Gel electrophoresis showing PCR detection of 1 kb fragment on 1.2% agarose gel. **M** is a size Marker, **+** plasmid pUC57 positive control,—negative water control, lanes **1–10** are putative transgenic plant lines, 11 and is a non-transgenic plant regenerated from somatic embryo.

The complete coding sequence of identified *MeFT1* was placed under control of the Cassava vein mosaic virus (CsVMV) promoter [27] and the *Agrobacterium tumefaciens* nopaline synthase (NOS) terminator sequence, and the combined 1,333 bp sequence was synthesized (GenScript, Piscataway, NJ) (Fig 1C). Plasmid pUC57 harboring the *MeFT1* expression cassette was double digested with restriction enzymes *Asc1* and *Sbf1* and the gel-purified fragment ligated into vector p5000 [28] pre-digested with the same restriction enzymes. Integrity of the final vector, named p8609, was confirmed by restriction digestion and sequencing. Both p8609 and empty vector p5000 (EV) gene constructs were transformed by electroporation into *Agrobacterium* strain LBA4404.

### Genetic transformation and plant regeneration

Young leaf explants were isolated from *in-vitro* plantlets of variety 60444 and cultured for a minimum of 30 days on Murashige and Skoog (MS) ([29]) media supplemented with 50 μM picloram to initiate production of organized embryogenic structures (OES) [27]. OES was isolated, fragmented and sub-cultured onto Gresshoff and Doy (GD) basal medium supplemented with 50 μM picloram (GD2 50P) for production of friable embryogenic callus (FEC). *Agrobacterium* carrying plasmids p8609, empty vectors p5000 and green fluorescent protein (GFP) controls ([28]) were used for co-cultivation of 18–21 day-old FEC tissues. Two independent transformation experiments each with three replications per construct were performed. Putative transgenic tissues were cultured on GD2 50P medium supplemented with 250 mg/L carbinicillin and 27.5 μM paromomycin for 21 days. Actively growing FEC colonies were sub-cultured onto regeneration medium consisting of MS2 medium containing 45 μM paromomycin and naphthaleneacetic acid (NAA) at 5 μM and 0.5 μM for 21 days each. Green cotyledon-stage embryos were transferred to antibiotic free MS2 medium supplemented with 2 μM 6-benzylaminopurine and cultured for four weeks. All cultures were maintained at 28 ±1°C, light and dark periods of 16/8, using 75 μmol m^−^2 s^−1^ fluorescent lamps [27].

### Plant establishment in the greenhouse and grafting

*In-vitro* shoots were sub-cultured onto MS2 medium solidified with 2.2 g/l phytogel 21–28 days prior to transferring to the greenhouse. Gel was gently washed off the roots and plantlets transferred to 3-inch pots filled with Fafard 51 soilless potting medium (Conrad Fafard, Inc., Agawam, MA, USA). The *in-vitro* plantlets were transplanted to soil at the 4–6 leaf stage. The transgenic and non-transgenic plants were hardened at 100% humidity for 7–10 days on a mist bench, and then moved to the open bench and grown at a temperature of 26/25°C (day/night) with 60%–90% relative humidity. A weekly Jack’s Professional fertilizer regime was maintained [26]. The flowering phenotype was evaluated under day neutral conditions of 12-hour light /12-hour dark photoperiod at 28°C and average relative humidity of 70% [26] over 120–200 day duration.

Between five and ten clonal plants per transgenic line were transplanted to soil and transferred to the greenhouse for evaluation of flowering phenotype. Hardened plants were grown for at least 120 days to produce stem cuttings. Semi-woody stem cuttings consisting of five nodes were harvested from the middle portion of the stem and established in 6-inch pots containing Fafard 51 potting medium to study clonal transmission of induced flowering and effect of *MeFT1* overexpression on storage root development.

Scions obtained from non-transgenic hardened plants of cultivars 60444 and TME 204 were cleft grafted onto transgenic rootstocks according to Souza *et al*. [7]. Briefly, a ‘V’ shaped incision aligned to the cambial region was made in the stem of 28-days old *FT* overexpressing transgenic rootstocks from tissue culture. A scion carrying matching ‘V’ configuration was inserted into the scion/rootstock junction and wrapped with parafilm. Non-transgenic 60444 rootstocks from tissue culture was cleft grafted with scions of both cultivars as controls. A total of 15 grafts per cultivar were performed. Grafted plants were maintained in a humidity chamber (80–100% humidity) for 10 days before moving to the open greenhouse bench and assessed for flowering.

### Tissue sampling and nucleic acid extraction

A total of 27 biologically independent samples were taken from *in-vitro* regenerated plantlets and from greenhouse grown plants between 30 and 200 days after planting (DAP) in soil. All samples were collated at the same time of day and comprised expanded leaves, shoot apexes, inflorescence and peeled storage roots. Tissues were wrapped in aluminium foil, instantly frozen in liquid nitrogen and stored at -80°C. A maximum of 0.1 g frozen tissue was transferred into 2 ml screwcap tubes each containing one ceramic bead and ground into fine powder using a Fast-Prep 24 (https://www.mpbio.com/). Genomic DNA (gDNA) and total RNA were extracted using the cetyltrimethylammonium bromide (CTAB) protocol [30]. RNA was removed from gDNA by RNase treatment using the DNase-free kit (www.roche.com), while DNA was removed from total RNA by treating with DNase using the DNA free Turbo kit (www.thermofisher.com/). Final concentration of RNA and DNA was determined with NanoDrop 2000c Spectrophotometer (www.thermofisher.com/) before gDNA and total RNA was preserved at -20°C and -80°C respectively.

### Confirmation of transgenic plants

Approximately 0.05 g of fresh leaf sample was obtained from putatively transgenic *in-vitro* plantlets, and crushed to detect neomycin phosphotransferase II (NPTII) using ImmunoStrips following manufacturer’s instructions (http://www.agdia.com/). PCR amplification of the CsVMV promoter and NOS terminator region of T-DNA cassette was carried out in a 25 μl reaction volume containing 100 ng gDNA, 12.5 μl high fidelity Phusion PCR master mix, 8 μl nucleic acid free DEPC treated water, 1.25 μl each of 10 μM forward and reverse primers (S1 Table). PCR conditions were; 98°C for 5 min, 35 cycles of 98°C for 10 s, 69°C for 20 s, 72°C for 30 s, followed by final extension at 72°C for 5 min [31]. Presence of the T-DNA cassette and size of the amplified fragment was visualized on GelRed^™^ stained (www.sycamorelifesciences.com) 1.2% agarose gel under UV light in Gel Doc XR+ system (www.bio-rad.com).

### Quantification of gene expression

Two micrograms of total RNA was used for complementary DNA (cDNA) synthesis using SuperScript III first strand synthesis kit (www.thermofisher.com/). Gene specific primers (95–200 bp) designed with Primer3 software (version 0.4.0) [32] were validated [33] and the best primer pair used for routine RT-qPCR analysis (S1 Table). Relative transcript abundance of *MeFT1* and downstream floral meristem identity genes *MeFD*, *MeAP1*, *MeSOC1* and *MeLFY1* were quantified by RT-qPCR using a CFX384 Touch Real-Time PCR Detection System (www.bio-rad.com). A 10 μl reaction mixture containing approximately 100 ng of cDNA template, SsoAdvanced universal SBGR Green SuperMix (Bio-Rad) as RT-qPCR master mix and 0.5 μm forward and reverse primers was used. Reaction conditions were; 95°C for 3 min; 39 cycles of 95°C for 10 s, 61°C for 10 s, 72°C for 20 s; followed by melting at 95°C, and melting curve analysis between 65°C and 95°C in 0.5°C increments for 0.05 s. Two plate readings were taken at extension (72°C) and melting curve analysis (65°C) stages. Two technical replicates were maintained for each triplicate biological sample. Data were analysed using the 2^−ΔΔCt^ formula [34] to determine expression levels of target genes relative to cassava reference genes *PP2A* [35]. Relative expression values from three different qPCR reactions were averaged to obtain final transcript abundance.

### Data collection and analysis

Plants were scored for onset of flowering starting one week after transfer to soil. Based on Ceballos *et al*. [4], a plant was assessed to have branched or forked when the apical shoot initiated two or more lateral shoots. Flowering was defined by presence of such branches whether or not an inflorescence containing male and female flowers appeared. Since cassava flowers multiple times in a season leading to production of several tiers of branches or flowers on the same plants, data was collected to determine number of days to first, second, third, fourth and fifth flowering events (DTF1-DTF5). Similarly, the number of nodes produced before commitment to first, second, third, fourth and fifth flowering events (NTF1-NTF5) were recorded. The number of male and female flowers produced at each flowering cycle was assessed and number of storage roots produced per plant line counted at harvest after approximately 200 days growth in the greenhouse. Prior to harvesting, the stem width at 10 and 20 nodes above the soil line was measured and the number of stems produced per plant counted. Shoot tissues (leaves, stems) and storage roots were oven dried (www.darwinchambers.com) at 37°C for 21 days to determine dry weight biomass. Whole plant dry weight was calculated by adding values for storage roots and above ground biomass. Two-way Student’s t test was performed with R software (version 3.4) to compare means for transgenic and non-transgenic controls and ggplot2 used to draw box and whisker plots [36,37].

## Results

### Candidate gene selection

A BLAST search of the cassava genome database in Phytozome 11 (version 4.1) with Arabidopsis *FT* (AT1G65480.1) nucleotide sequence identified 10 *FT* family genes encoding PEBP [21]. We selected *MeFT1* for gain of function studies based on its similarity with *AtFT*, the latter of which triggered early flowering when overexpressed in cassava [20]. The genomic sequence of *MeFT1* was seen to consist of 1,987 nucleotides, within which there are four exons and three introns (Fig 1A) that code for 175 amino acids. Pair-wise sequence alignment revealed that both *AtFT* and *MeFT1* share amino acid Y (tyrosine at position 86) and several identical amino acids conserved in segments B and C (Fig 1B) that are known to a play a critical role in regulation of flowering [38]. The synthesized *MeFT1* shares 76.4% sequence homology with *AtFT* and is identical to the *MeFT1* in '60444 at the amino acid level.

### Production and molecular analysis of plants

Thirty-five putatively transgenic embryogenic callus lines were produced using construct p8609 in which *MeFT1* was placed under control of the strong constitutive CsVMV promoter. Of these, 17 lines died after transfer to MS2 2BAP germination media, while somatic embryos from a further eight lines failed to germinate despite repeated sub-culture on medium containing BAP. Ten lines produced healthy somatic embryos and germinated to produce whole plants *in-vitro*. Embryos from EV and non-transgenic controls regenerated to produce plants as expected [26] (Table 1).

**Table 1:** Production of transgenic plant lines in cv. 60444 and flowering rates under *in-vitro* conditions.

Construct*	Number of embryogenic callus lines produced	Number of plant lines regenerated	Number of plant lines flowering *in-vitro*	Number of plant lines flowering that survived *in-vitro*	Number of plant lines established in greenhouse
*MeFT1* (p8609)	35	10	10/10	2/10	2/10
EV (p5000)	8	2	0/2	0/2	na
Control	10	3	0/3	0/3	1/3

^*^MeFT1 = overexpressing transgenic plant lines, **EV** = transgenic empty vector controls, **Control**–embryogenic tissues were not exposed to Agrobacterium.

All 10 putative *MeFT1* transgenic plant lines recovered tested positive for NPTII activity by ImmunoStrip analysis and displayed the expected 1000 bp signal after PCR amplification of the *MeFT1* expression cassette (Fig 1C).

### Overexpression of *MeFT1* produced precocious *in-vitro* flowering

**Fig 2:**
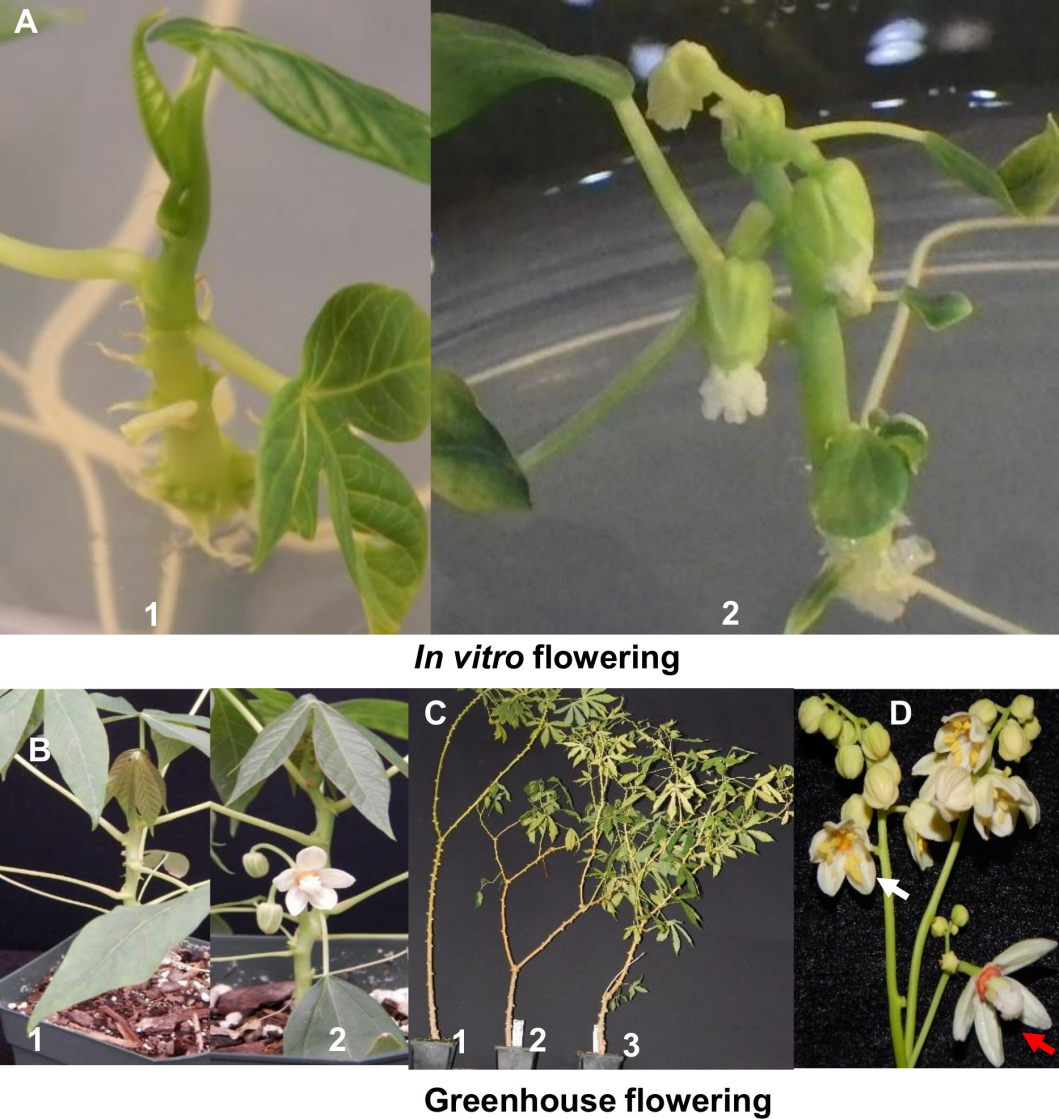
Flowering in transgenic 60444 plants expressing *MeFT1* under *in vitro* and greenhouse conditions. (**A**)Transgenic plant line MeFT1-2 flowering *in vitro* while growing on MS-based medium (A-2). (**B**) MeFT1-1 plant line flowering 14 days after transfer to soil (C-2). Non-flowering plants are non-transgenic controls regenerated from somatic embryos (left) (A-1, C-2). (**C**) Branching phenotype in repetitively flowering transgenic plant lines (C-2, C-3) compared to control 180 days after planting (C-1). (**D**) Inflorescence produced from stake derived plant line MeFT1-1 showing numerous male flowers above opened female flowers (red arrow) 90 DAP, white arrow indicates open male flower about 20 days after emergence in transgenic plant line MeFT1-2.

Flowering was observed *in-vitro* in 10/10 (100%) plant lines transgenic for *MeFT1*, starting 14 days after transfer to fresh MS2 medium. Flower buds were produced from both apical and auxiliary meristems with flowers aborting within 21 days. Eight of the 10 flowering plant lines (80%) died approximately 21 days after commencement of flowering. The remaining two plant lines, named MeFT1*–*1 and MeFT1-2 survived and could be routinely micropropagated at 90-day intervals on MS2 medium. In both of these transgenic events, *in-vitro* flowering resumed approximately 21 days after sub-culture onto fresh MS2 medium (Fig 2A–2). No flowering *in-vitro* was observed at any time from plants regenerated from EV lines or somatic embryo (SE) derived, non-transgenic controls (Fig 2A–1).

### *MeFT1* expression triggered flowering across clonal propagation cycles in the greenhouse

**Fig 3:**
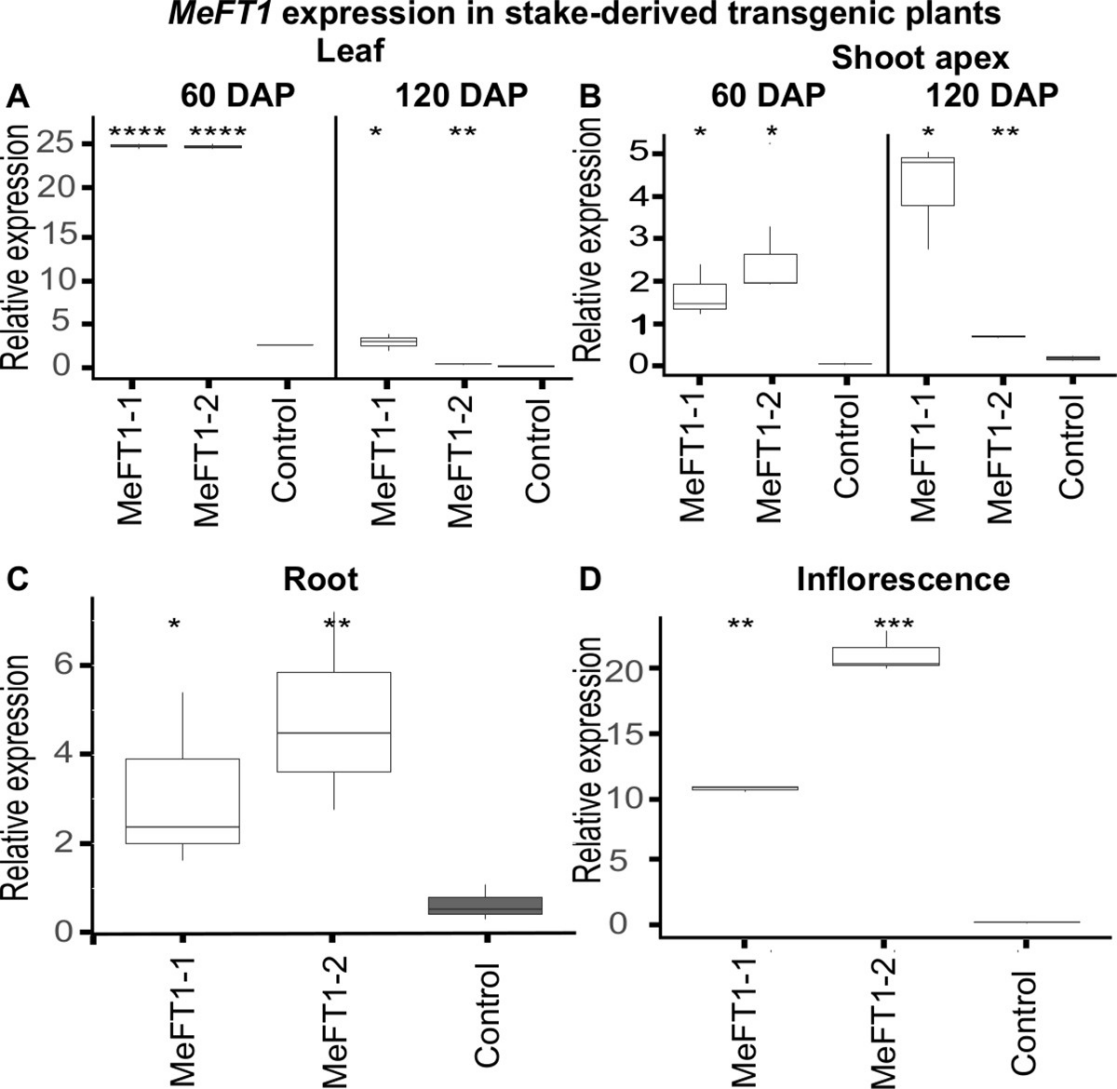
RT-qPCR expression profiling of *MeFT1* in multiple tissues of transgenic plant lines MeFT1-1, MeFT1-2 and somatic embryo-derived non-transgenic control. Relative mRNA levels of MeFT1 in (**A**) leaf at 60 and 120 days after planting (DAP), (**B**) shoot apex at 150 DAP, (**C**) root and (**D**) inflorescence at 210 DAP respectively. Data shown is for three independent RT-qPCR reactions each comprising three biological samples and two technical replications per tissue type. Two-way Student’s t test was performed to compare means of transgenic and non-transgenic embryo derived controls. *P ≤ 0.05; **P ≤ 0.01; ***P ≤ 0.001, ****P ≤ 0.0001. ggplot2 of R software was used to construct box plots. The middle line in the box shows the median value. Data ranging from smallest value to lower quartile represents lower whisker while values between upper quartile and largest correspond to upper whisker. Values below and above median represent lower and upper quartile of roughly 1.5IQR each.

Transgenic plant lines MeFT1-1 and MeFT1-2 were hardened along with the somatic embryo (SE) -derived non-transgenic controls and established in the greenhouse. RT-qPCR analysis of *MeFT1* transcript abundance demonstrated a highly significant (*P≤0*.*001*) increase in *MeFT1* transcript levels in leaves at 60 days after establishment in soil, with a 10-fold increase registered by both transgenic plant lines compared to leaves of non-transgenic control plants (Fig 1). Transgenic inflorescence tissues also expressed significantly higher *MeFT1* mRNA (*P≤0*.*001)* for both transgenic plant lines than the controls (Fig 3D). Transcript levels of *MeFT1* in the shoot apex and storage roots of the two transgenic plant lines were higher at significance levels *P≤0*.*05* to *P≤0*.*001* compared to SE-derived controls (Fig 3B and 3C).

**Fig 4:**
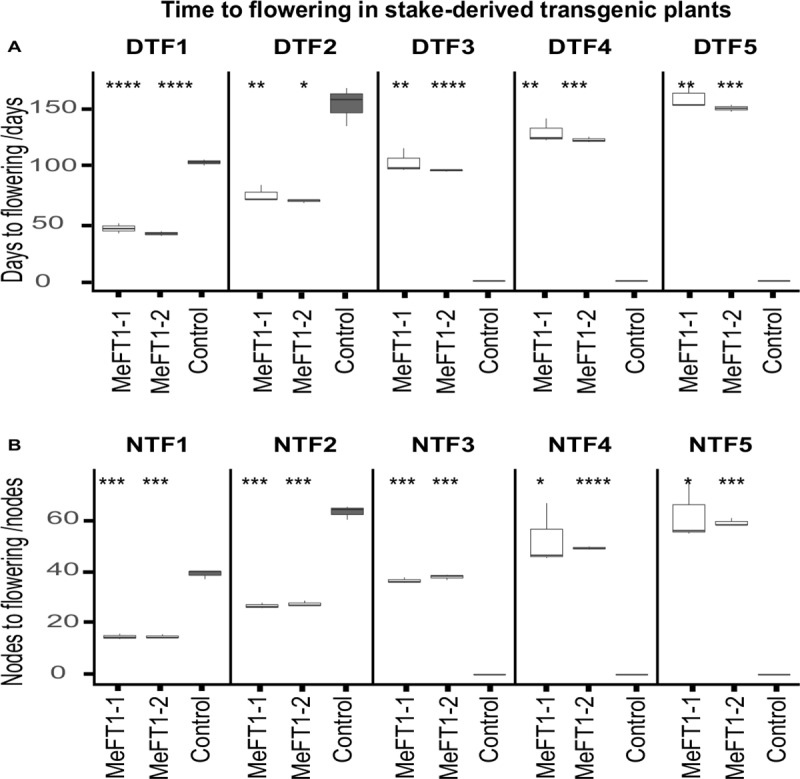
Flowering time in *MeFT1* overexpressing and somatic embryo-derived control plant lines of cultivar 60444. (**A**) Days to first, second, third, fourth and fifth (DTF 1–5) flowering cycles, (**B**) number of nodes produced by plants prior to the first, second, third, fourth and fifth (NTF 1–5) flowering cycles. MeFT1-1 and MeFT1-2 are transgenic plant lines while SE is a somatic embryo-derived non-transgenic control. n = 30 independent plants per plant line. Two-way Student’s t test was performed to compare means of transgenic and non-transgenic embryo derived controls. *P ≤ 0.05; **P ≤ 0.01; ***P ≤ 0.001, ****P ≤ 0.0001. ggplot2 of R software was used to construct box plots. The middle line in the box shows the median value. Data ranging from smallest value to lower quartile represents lower whisker while values between upper quartile and largest correspond to upper whisker. Values below and above median represent lower and upper quartile of roughly 1.5IQR each.

Within 14 days after establishment in soil, transgenic plants of MeFT1-1 and MeFT1-2 produced flower buds. Both lines recorded similar number of days to flowering (DTF) and nodes to flowering (NFT) across four flowering cycles over a 120-day observation period. Over the same time period no flowering was observed from the SE-derived control plants (Fig 2B–1). After 120 days growth in the greenhouse, stake cuttings were obtained from the transgenic plant lines and SE-derived controls and replanted to determine if overexpression of *MeFT1* induced flowering across the vegetative cropping cycle, and if associated effects were present in storage root development. First flowering was observed in all 30 clonal replicates of each transgenic plant line between 20 and 50 days after planting stem cuttings. Both transgenic plant lines behaved similarly, recording five flowering cycles, forking each time to generate two or three branches across the 200-day observation period. SE-derived control plants flowered twice at approximately 90 days after planting, by which time plants from both transgenic lines had undergone three flowering cycles. Significant differences were therefore observed for average DTF and NTF at (*P≤0*.*01*) and (*P≤0*.*001*) respectively between transgenic and SE-derived control plants. For example, average DTF1 for plant lines MeFT1-1 and MeFT1-2 were 45.5 and 41.0 days compared to 102.7 days in SE-derived control plants respectively (Fig 4A) and average NTF1 was 14.7 nodes for both transgenic plant lines versus 39 nodes in SE-derived control plants (Fig 4A and 4B).

**Fig 5:**
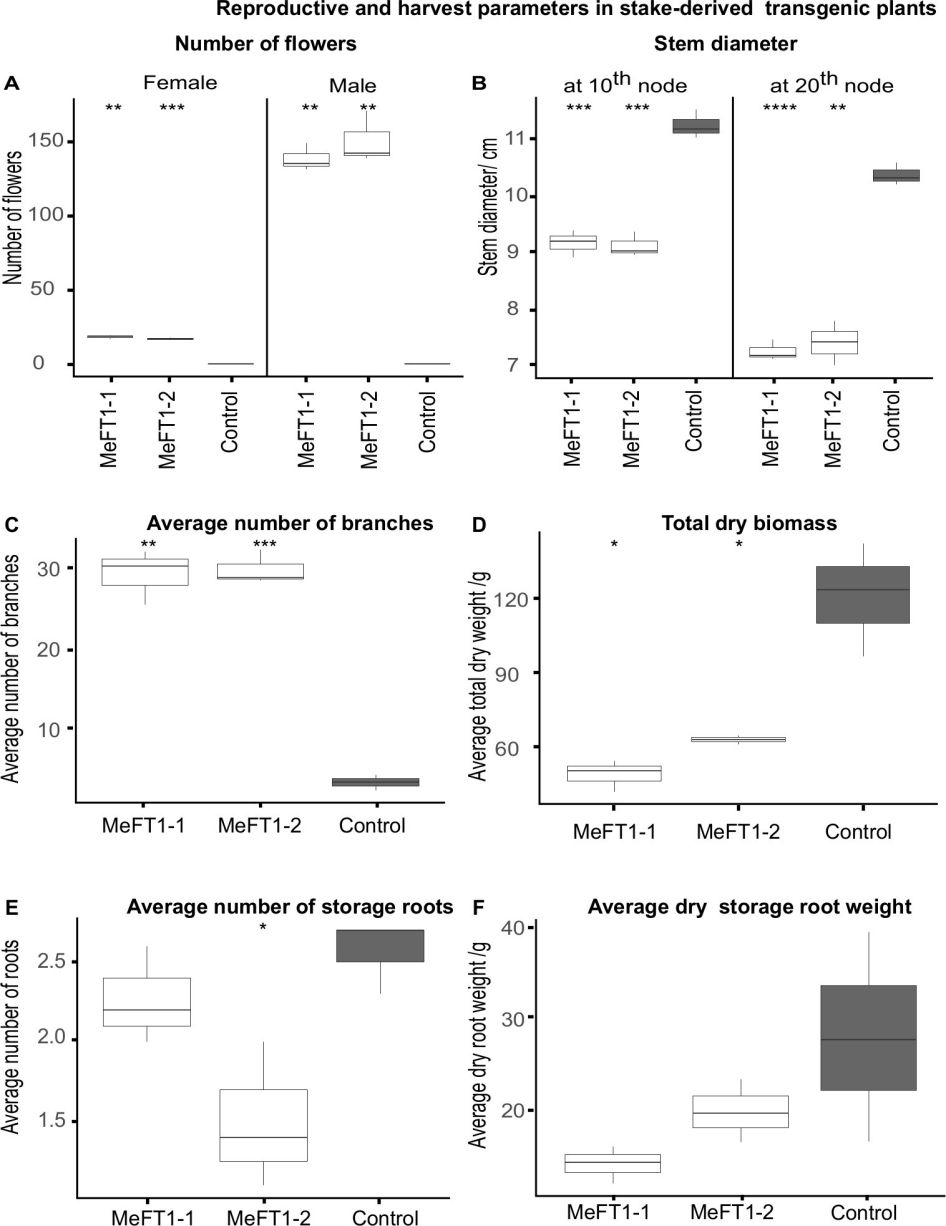
Growth and harvest parameters of *MeFT1* overexpressing and somatic embryo-derived non-transgenic control plant lines assessed at 200-day growth period in the greenhouse. (**A**) Average number of male and female flowers, (**B**) average number of branches produced per plant, (**C**) average stem thickness, (**D**) total dry weight of shoot and storage roots, (E) average number of storage roots per plant and (F) average storage root weight. n = 30 independent plants per plant line in all cases except for (C) in which n = 10 independent plants. Two-way Student’s t test was performed to compare means of transgenic and non-transgenic embryo derived controls. *P ≤ 0.05; **P ≤ 0.01; ***P ≤ 0.001, ****P ≤ 0.0001. ggplot2 of R software was used to construct box plots. The middle line in the box shows the median value. Data ranging from smallest value to lower quartile represents lower whisker while values between upper quartile and largest correspond to upper whisker. Values below and above median represent lower and upper quartile of roughly 1.5IQR each.

*MeFT1* expressing plant lines produced well-formed male and female flowers carrying distinct anthers and stigmas which were retained for at least 20 days and attained full maturity (Fig 2D). In contrast, SE-derived controls produced flowers that aborted within 7 days after emergence (Fig 5A). Plants of MeFT1-1 and MeFT1-2 both produced significantly more male (average 144.1) than female (average 17.6) flowers per plant. Since branching is a precursor to flowering in cassava, we counted and compared the number of branches produced by each plant over time. Constitutive expression of *MeFT1* stimulated significant (*P≤0*.*05*) and highly significant (*P≤0*.*001*) branching in transgenic plant lines compared to the SE-derived controls, translating into a bushy architecture in both transgenic plants lines (Fig 5C). The SE-derived control plants produced 26 fewer branches per plant and possessed thicker stems than the two transgenic counterparts (Fig 5B).

In order to test if *MeFT1* engineered flowering was graft transmissible, 15 hardened non-transgenic plants each of the lesser flowering cv. TME 204 and 60444 were cleft grafted onto transgenic rootstocks of transgenic plant line MeFT1-2. No flowering was observed in any plants of either cultivar whether grafted onto transgenic or non-transgenic rootstocks over a 180 day observation period. This was the case irrespective of presence or absence of three expanded leaves retained below the graft union.

The effect of increased *MeFT1* expression on storage root development was examined in stake-derived transgenic and SE-derived plants harvested after 200 days growth in the greenhouse. The average total dry weight of transgenic plant lines was significantly lower (*P≤0*.*05*) than those from SE-derived control plants. Both transgenic plant lines produced fewer storage roots per plant than SE-derived controls (Fig 5D and 5E). Average dry root weight of the two transgenic plant lines was lower but not significantly different in relation to values for SE-derived control plants. The median dry storage root weights for transgenic plant lines MeFT1-1 and MeFT1-2 are 15.0 g and 19.0 g respectively compared to 27.5 g for the control (Fig 5F).

### Upregulation of *MeFT1* is associated with increased expression of floral meristem identity genes in transgenic plants

**Fig 6:**
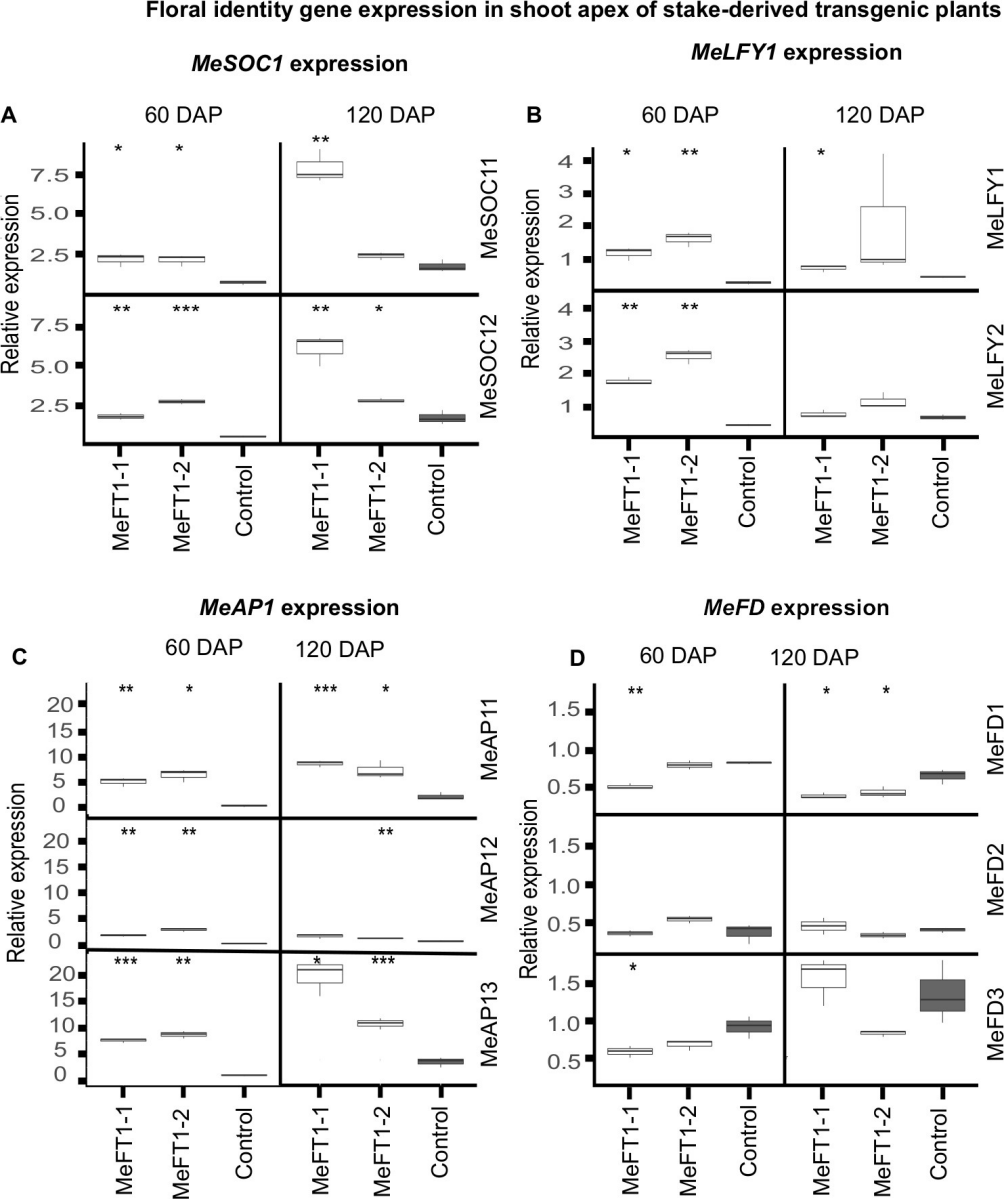
RT-qPCR expression profiling of floral identity genes in shoot apex of transgenic plant lines MeFT1-1, MeFT1-2 and somatic embryo-derived non-transgenic controls. Relative transcript abundance of (**A**) Apetala 1 (*MeAP1*), (**B**) Leafy (*MeLFY*), (**C**) Flowering Locus D (*MeFD*), (**D**) Suppressor of Expression of Constans 1 (*MeSOC1*) at 60 and 120 DAP. Data is shown for three independent RT-qPCR reactions each comprising three biological samples and two technical replications. Two-way Student’s t test was performed to compare means of transgenic and non-transgenic embryo derived controls. *P ≤ 0.05; **P ≤ 0.01; ***P ≤ 0.001. ggplot2 of R software was used to construct box plots. The middle line in the box shows the median value. Data ranging from smallest value to lower quartile represents lower whisker while values between upper quartile and largest correspond to upper whisker. Values below and above median represent lower and upper quartile of roughly 1.5IQR each.

Levels of mRNA expressed by downstream floral organ identity genes *MeAP1*, *MeLFY*, *MeSOC1* and *MeFD* were assessed in the shoot apex of stake derived transgenic plants. Compared to SE-derived control plants, transcript levels for all *MeAP1*, *MeSOC1* and *MeLFY* ranged from not significant to highly significant (*P≤0*.*001*) in the shoot apex of both transgenic plant lines at 60 and 120 DAP. *MeAP12* and *MeLFY2* recorded insignificantly higher transcript values in transgenic plants compared to SE-derived control plants (Fig 6A–6C). The expression pattern of *MeFD* was found to be inconsistent at 60 and 120 DAP compared to the three floral meristem identity genes. With minor exceptions, the three *MeFD* genes recorded either similar or significantly lower (*P≤0*.*01*) transcript levels in transgenic plant lines compared to SE-derived control plants (Fig 6D).

## Discussion

Irregular flowering constrains cassava breeding programs, while knowledge of genes and pathways regulating flowering in this crop remains scarce. The present study identified 10 PEPB encoding *FT*-like genes in a manner similar to that reported earlier [21]. Among them, *MeFT1* (Manes.12G001600.1) was seen to share critical conserved amino acids with Arabidopsis FT [38]. High expression of *MeFT1* was reported in the prolific flowering cassava accession IBA980002, but the expression data could not be linked to flower induction function [21]. Here we report production of inflorescences from plants lines transgenic for expression of *MeFT1*. These findings correlate with heterologous overexpression of *AtFT* in cassava [20,25] and represents first evidence for flower induction by manipulation of a native *FT*-like gene in cassava. A consequence of such rapid transition to the flower phase is commitment of meristems to produce inflorescences and termination of vegetative growth. As a result, only two out of ten *MeFT1* expressing transgenic plant lines survived *in-vitro* flowering. In a similar manner, profusely flowering wheat and Brachypodium plants transgenic for *FT1* exhibited diminished shoot biomass and problematic establishment in soil [39].

Cassava is vegetatively propagated via stem cuttings. Accelerated flowering in two transgenic cassava plant lines was maintained through vegetative propagation cycles under greenhouse conditions. Plants exhibited up to five flowering events with branches produced at each forking over a 200-day period, while non-transgenic controls flowered twice. Previous reports recorded positive association between *AtFT* overexpression in cassava and production of mature male and female flowers. In the current study, endogenous *MeFT1* overexpression produced mature male and female flowers with well-developed anthers and stigmas before undergoing senescence after 20 days. This contrasted with non-transgenic controls plants whose flowers did not mature and aborted within 7 days of emergence (Fig 2C). This further confirms that *MeFT1* is involved in flower stimulation and development. Compared to SE-derived control counterparts, transgenic cassava plants reported here produced a bushy phenotype (Fig 5C), confirming earlier reports that modifying expression of *FT*-like genes impacts plant architecture [12,40].

Flower induction by overexpression of *FT* genes is known to be graft transmissible in other species and has been exploited to trigger flowering in non-transgenic scions as a strategy to shorten the breeding cycle ([41–43]). Our attempts induce flowering by graft transmission of *MeFT1* was unsuccessful. After grafting, leaves below the graft union often undergo senescence and abscise [27], thus reducing or eliminating the supply of MeFT1 protein. Loss of leaves below the graft union was also observed in the present study after grafting. This could explain why transgenically expressed *MeFT1* is not transmitted, or the amount of MeFT1 protein transported across the graft union is insufficient to trigger flowering in the scion. If true, this represents a technical limitation which could be addressed by use of larger, more mature transgenic rootstocks carrying more leaves onto which scions would be grafted. Possibly, this is why successful graft transmissible flowering and fruit production was achieved in cassava under field conditions, where plants would be more robust compared to those grown under contained conditions. Alternatively, additional signaling molecules may be required to initiate flowering in cassava. For example, in blueberry, graft transmissible flowering was reported following grafting of non-transgenic scions onto transgenic rootstocks overexpressing *FT*. This success was attributed to graft transmission of *VcFT* plus phytohormones ([44]). Further investigation is required to assess if a similar process is functioning in cassava.

Increased expression of *FT* has been associated with improved storage organ development in transgenic potato and onion [14,18]. However, no such positive correlation was observed in the present study. Indeed, the opposite occurred, with both transgenic plant lines recording significantly (P≤0.05) fewer storage roots and lower average storage root weight than non-transgenic controls (Fig 4D–4F). The inverse relationship between rapid flowering and storage root development recorded here and findings in potato and onion might be because storage organs in potato and onion are adapted shoot structures, while in cassava the storage organ is a genuine root. Under field conditions, cassava cultivars displaying delayed flowering and associated prolific branching have been reported to exhibit higher storage root yields [45,46]. Recent studies with transgenic cassava plants of cv. 60444 expressing *AtFT* also recorded a negative correlation between robust flowering and cassava storage root production [20]. Combined, this evidence indicates that overexpression of *FT* is a strategy unlikely to enhance storage root production in cassava. Conversely, suppression of *FT* may offer a route to adapt shoot architecture and enhanced storage root yields in floriferous cassava cultivars.

Relating transcript abundance of *FT*-like genes with phenotype is essential for functional validation. In this study, RT-qPCR-based quantification revealed upregulation of *MeFT1* mRNA levels in leaf, storage roots, inflorescence and the shoot apex of transgenic plants compared to SE-derived controls plants (Fig 3), including a 10-fold increase in *MeFT1* mRNA levels in leaves. This finding further validates the role of *MeFT1* in flower induction in cassava. *MeFT1* upregulation in the transgenic plants described here was also associated with increased transcript abundance of downstream floral meristem identity genes *MeAP1*, *MeSOC1* and *MeLFY* (Fig 6). Correlating flowering in cassava with increased expression of native *MeFT1* and floral meristem identity genes supports the hypothesis that the apical shoot of cassava has floral meristem identity genes that associate with *FT* genes to trigger flowering [20]. These mRNA profiles match findings in kiwifruit and jatropha expressing *AcFT1*, *AcFT2* and *JcFT* transgenes that resulted in upregulation of floral identify genes *LFY*, *SOC1* and *AP1* [18,19]. Based on these data, we propose that *MeFT1* induces flowering in cassava by positively regulating expression of floral meristem identity genes *MeAP1*, *MeSOC1* and *MeLFY*. These genes therefore present interesting additional targets for future manipulation of flowering in cassava.

In conclusion, transgenic overexpression of *MeFT1* resulted in production of early and mature flowers that exhibited extended retention time under *in-vitro* and greenhouse environments. Upregulation of native *MeFT1* was also associated with elevated expression of downstream floral meristem identity genes *MeAP1*, *MeLFY* and *MeSOC1* in tissues at different developmental stages. This supports *MeFT1* as a flower inducer in cassava and suggests further studies designed to validate roles of *TFL1*-related floral suppressor genes using transgenic and genome editing approaches.

## Supporting information

S1 Table List of primers used for molecular detection and RT-qPCR analysis. (PDF)

S1 Fig Gel electrophoresis picture showing PCR detection of 1 kb fragment on 1.2% agarose gel. **M** is a size Marker, **+** plasmid (pUC57) positive control,—negative water control, lanes **1–10** are putative transgenic plant lines, 11 and is a non-transgenic plant regenerated from somatic embryo.
(TIF)
