# Correction: Transgenic overexpression of endogenous FLOWERING LOCUS T-like gene *MeFT1* produces early flowering in cassava

**DOI:** 10.1371/journal.pone.0231232

**Published:** 2020-03-26

**Authors:** The PLOS ONE Staff

The second author’s name in incorrect. The correct name is: Getu Beyene. The correct citation is: Odipio J, Beyene G, Chauhan RD, Alicai T, Bart R, et al. (2020) Transgenic overexpression of endogenous FLOWERING LOCUS T-like gene *MeFT1* produces early flowering in cassava. PLoS ONE 15(1): e0227199. https://doi.org/10.1371/journal.pone.0227199.

The publisher apologizes for the error.
