# Supplementary material for: Transgenic overexpression of endogenous FLOWERING LOCUS T-like gene MeFT1 produces early flowering in cassava
Source: PLoS One. 2020 Jan 28;15(1):e0227199. doi: 10.1371/journal.pone.0227199 (PMC6986757; doi:10.1371/journal.pone.0227199)
Supplement: S1 Table — (PDF) [file pone.0227199.s001.pdf]

S1 Table 1: Primers used for molecular detection and RT-qPCR analysis

Primer number	Primer sequence	Targeted gene	Purpose
1	CACATGAGCGAAACCCTATAGGAACCC	p5000 vector	Molecular detection
2	GCACTGACGACAACAATGAAAAGA		
3	5'-TCGGCAATTAGGAAGACA-3'	*MeFT1*	RT-qPCR
4	5'-GTAAACAGCGGCAACGGGC-3'		
5	5'-GCATAGAGAACGCGACAAGCAGG-3'	*MeSOC11*	RT-qPCR
6	5'-GGGCAACCTCGGCATCGCAA-3'		
7	5'-AGGCGCTGGGTTCTGGTT-3'	*MeSOC12*	RT-qPCR
8	5'-ATGCAGGAGACAATTGAACG-3'		
9	5'-TCAGGTGTTCAGGTACGCC-3'	*MeLFY1*	RT-qPCR
10	5'-CTCTCCTCAGTGCATTGG-3'		
11	5'-GAAGGCAGGAGCAAGCTAC-3'	*MeLFY2*	RT-qPCR
12	5'-ATGCCTGTCTCCACGCTCCA-3'		
13	5'-TTGTTGAAGAAAGCCCATGAG-3'	*MeAP11*	RT-qPCR
14	5'-GTAGAGTATTCAAAGAGCTTCC-3'		
15	5'-CAAGCGGATCGAGAACAAG-3'	*MeAP12*	RT-qPCR
16	5'-TCAAAGCAACCTCAGCATC-3'		
17	5'-ATGGGGAGAGGTAGAGTTC-3'	*MeAP13*	RT-qPCR
18	5'-CCTCAGCATCGCAAAGCAC-3'		
19	5'-TCCGCCTTCCAATCTCCTC-3'	*MeFD1*	RT-qPCR
20	5'-ATGGCGAGTGGGAGCGAC-3'		
21	5'-ACTATGGAAGAGGTCTGG-3'	*MeFD2*	RT-qPCR
22	5'-GTTAGGGTTATGATGAGG-3'		
23	5'-GCGCCAAGAACTGCCACT-3'	*MeFD3*	RT-qPCR
24	5'-CGATGAAGAACCCAGAGCACC-3'		
25	5'-TGCAAGGCTCACACTTTCATC-3'	MePP2A	RT-qPCR
26	5'-CTGAGCGTAAAGCAGGGAAG-3'		
