# Supplementary figures and images for: Transgenic overexpression of endogenous FLOWERING LOCUS T-like gene MeFT1 produces early flowering in cassava
Source: PLoS One. 2020 Jan 28;15(1):e0227199. doi: 10.1371/journal.pone.0227199 (PMC6986757; doi:10.1371/journal.pone.0227199)

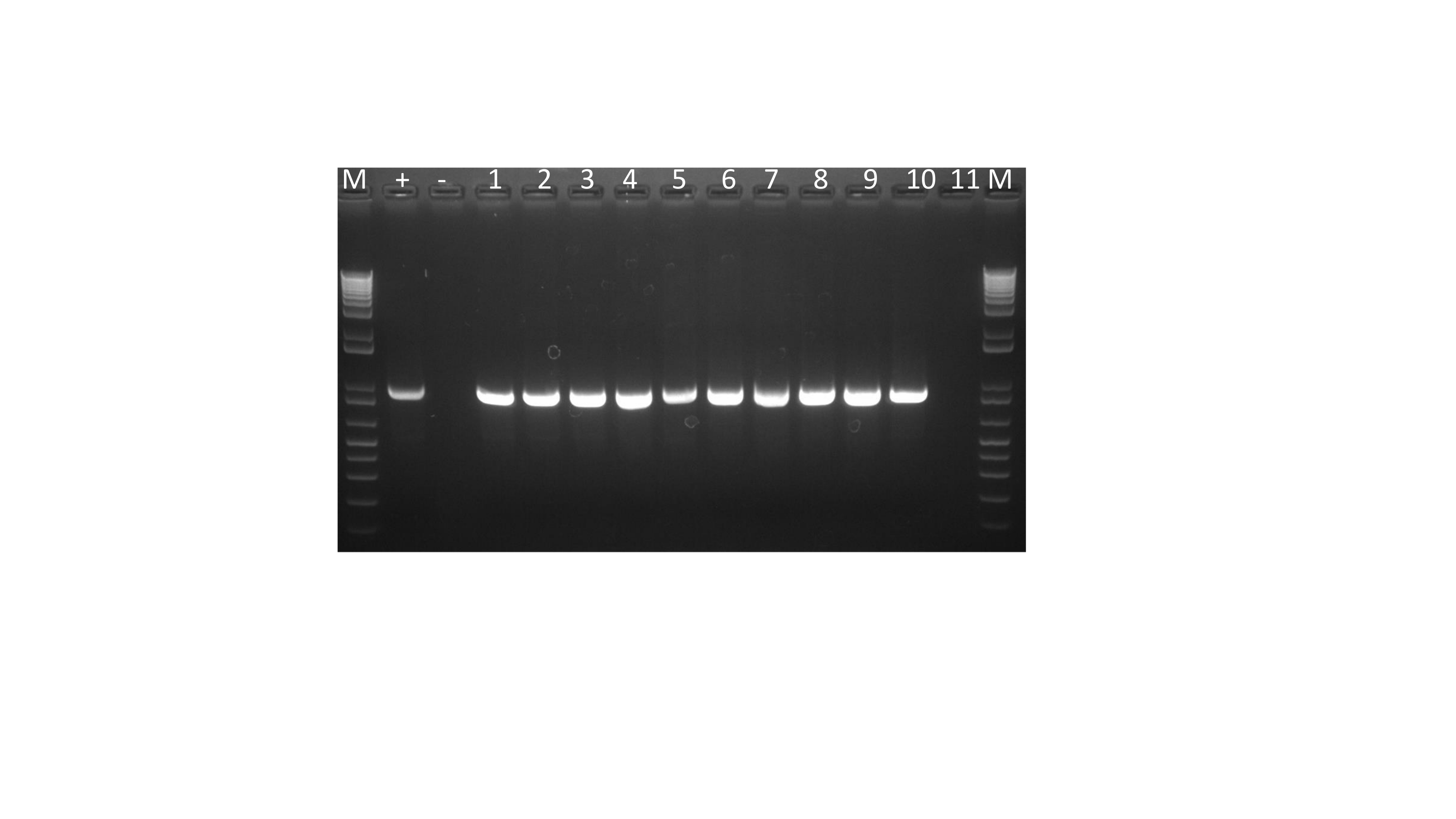

Supplement: S1 Fig — M is a size Marker, + plasmid (pUC57) positive control,—negative water control, lanes 1–10 are putative transgenic plant lines, 11 and is a non-transgenic plant regenerated from somatic embryo. (TIF) [file pone.0227199.s002.tif]
